# Growth disorders caused by variants in epigenetic regulators: progress and prospects

**DOI:** 10.3389/fendo.2024.1327378

**Published:** 2024-02-02

**Authors:** Julian C. Lui

**Affiliations:** Section on Growth and Development, National Institute of Child Health and Human Development, Bethesda, MD, United States

**Keywords:** bone elongation, endochondral ossification, chondrocytes, genetic diseases, HDAC4

## Abstract

Epigenetic modifications play an important role in regulation of transcription and gene expression. The molecular machinery governing epigenetic modifications, also known as epigenetic regulators, include non-coding RNA, chromatin remodelers, and enzymes or proteins responsible for binding, reading, writing and erasing DNA and histone modifications. Recent advancement in human genetics and high throughput sequencing technology have allowed the identification of causative variants, many of which are epigenetic regulators, for a wide variety of childhood growth disorders that include skeletal dysplasias, idiopathic short stature, and generalized overgrowth syndromes. In this review, we highlight the connection between epigenetic modifications, genetic variants in epigenetic regulators and childhood growth disorders being established over the past decade, discuss their insights into skeletal biology, and the potential of epidrugs as a new type of therapeutic intervention.

## Introduction

In the eukaryotic cell nucleus, double stranded DNA wraps around core histone proteins composed of an octamer of H2A, H2B, H3 and H4, forming the basic unit of chromatin called nucleosome. Nucleosomes are then further assembled in the cell nucleus into highly compacted structure of chromatin and chromosomes. In order for transcription factors to navigate the condense genetic material and access the individual promoter and gene body to initiate gene expression, localized loosening of chromatin structure is necessary, and this could be achieved by a combination of chromatin remodeling, changes of DNA methylation, and post-translational modifications of the core histones. These molecular changes that affect gene expression without altering the underlying DNA sequence is also known as epigenetic modifications (1).

**Figure 1:**
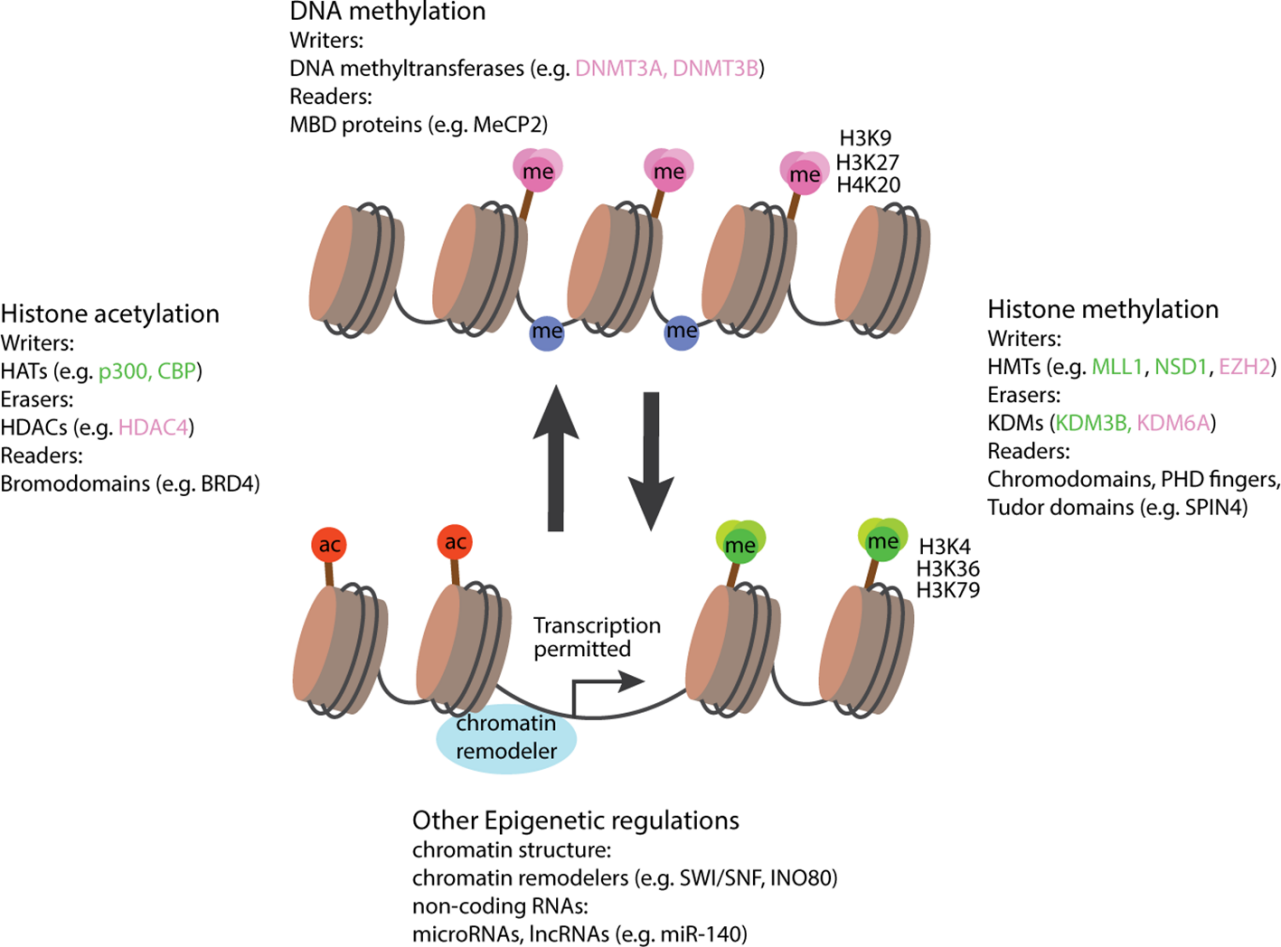
Schematic diagram illustrating the involvement of epigenetic regulators in activation and suppression of transcription. In general, modifications associated with gene activations include an open chromatin structure, histone acetylation, histone methylation at residue H3K4, H3K36, H3K79, and a loss of DNA methylation. Epigenetic regulators favoring these conditions are labeled in green. Modifications associated with gene suppression include a closed chromatin structure, histone deacetylation, histone methylation at H3K9, H3K27, H4K20, and a gain of DNA methylation. Epigenetic regulators favoring these conditions are labeled in pink. Epigenetic regulators not associated with either activation or suppression are labeled in black. HATs, histone acetyltransferases; HDACs, histone deacetylases; MBD, Methyl-CpG-binding domain; HMTs, histone methyltransferases; KDMs, histone demethylases, ac, acetyl group; me, methyl group. Right-angled arrow indicates active transcription.

Over the years, a large number of molecules has been identified to regulate gene expression via epigenetic mechanisms, molecules that could now be categorized into several classes of epigenetic regulators (2). For example, enzymes that add epigenetic modifications are referred to as epigenetic writers, which include DNA methyltransferases, histone acetyltransferases (HATs) and methyltransferases (KMTs), while enzymes that remove modifications are referred to as epigenetic erasers, such as histone deacetylases (HDACs) and demethylases (KDMs) (3). In addition, epigenetic readers are proteins, rather than enzymes, that recognize and bind to specific epigenetic modifications to modulate downstream transcriptional activity. And then there are epigenetic regulators that do not fall into this ternary classification of epigenetic writer-eraser-reader, and those include chromatin remodelers that affect chromatin accessibility by higher level structural changes, and non-coding RNA (ncRNA) that participate in post-transcriptional regulation of gene expression (**Figure 1**).

In the past 20 years, tremendous advancement in sequencing technology have led to a quantum leap in genetic diagnosis of Mendelian disorders. There are now more than 400 different genes associated with various types of skeletal dysplasia (4), and many more have been identified for childhood growth disorders that includes primordial dwarfism, idiopathic short stature and overgrowth syndromes. Although pathogenic variants of epigenetic regulators do not necessarily contribute to an outsized proportion to childhood growth disorders overall, they do offer some potential advantages for drug development, as implicated by the theoretical reversibility and plasticity of the epigenome. In this review, we aim to highlight the progresses being made in the past decade on childhood growth disorder implicated by epigenetic regulators (**Table 1**), address the challenges that remain, and discuss their potential insights into therapeutic development.

**Table 1:** List of epigenetic regulators implicated in human growth disorders.

Epigenetic modifications	Gene	Function	Human disorder (s)	Growth phenotype	Gene (s) or pathway(s) involved?
**Histone Acetylation**	(Writers)				
	P300	Histone acetyltransferase	Rubinstein-Taybi syndrome (OMIM 180849)	Short stature	SOX9,COL2A1
	CBP	Histone acetyltransferase	Menke-Hennekam syndrome (OMIM 618332)	Short stature	
	KAT6B	Histone acetyltransferase	SBBYS syndrome (OMIM 603736)	Short stature	
	(Erasers)				
	HDAC4	Histone deacetylase	BDMR syndrome (OMIM 600430)	Short stature	RUNX2, MEF2C
	HDAC8	Histone deacetylase	Cornelia de Lange Syndrome 5 (OMIM 300882)	Short stature	SMC3
**Histone Methylation**	**(Writers)**				
H3K4	KMT2D	Histone methyltransferase	Kabuki syndrome 1 (OMIM 147920)	Short stature	SHOX2, SOX9
H3K4	KMT2A	Histone methyltransferase	Wiedemann-Steiner Syndrome (OMIM 605130)	Short stature	
H3K36	NSD1	Histone methyltransferase	Sotos syndrome (OMIM 117550)	Overgrowth	SOX9, Hif1α
H3K36	SETD2	Histone methyltransferase	Luscan-Lumish syndrome (OMIM 616831)	Overgrowth	
H3K27	EZH2	PRC2 complex component	Weaver syndrome (OMIM 277590)	Overgrowth	IGF signaling
H3K27	EED	PRC2 complex component	Cohen-Gibson syndrome (OMIM 617561)	Overgrowth	WNT, TGF-β
H3K27	SUZ12	PRC2 complex component	Imagawa-Matsumoto syndrome (OMIM 618786)	Overgrowth	
	**(Erasers)**				
H3K9	KDM3B	Histone demethylase	Diets Jongmans syndrome (OMIM 618846)	Short stature	
H3K4	KDM6A	Histone demethylase	Kabuki syndrome 2 (OMIM 300867)	Short stature	
	**(Readers)**				
H3K4	SPIN4	Binds methylated histones	Lui-Jee-Baron syndrome (OMIM 301114)	Overgrowth	WNT signaling
**DNA methylation**	**(Writers)**				
	DNMT3A	DNA methyltransferase	Tatton Brown Rahman syndrome (OMIM 615879)	Overgrowth	
	**(Readers)**				
	MECP2	Binds methylated DNA	Rett Syndrome (OMIM 312750)	Short stature	
**Others**	CHD8	Chromatin remodeler	IDDAM syndrome (OMIM 615032)	Overgrowth	
	miR-140	Non-coding RNA	Spondyloepiphyseal dysplasia (OMIM 618618)	Short stature	BMPs, Hif1α

## Childhood bone growth and the epiphyseal growth plate

Genetic disorders affecting childhood growth can be manifested in many different shapes and forms, but they all inevitably impact a child’s normal skeletal development, usually in the form of short stature, tall stature/overgrowth, and in some cases that more severely affect bone development, skeletal dysplasias. Childhood bone growth is driven by a process called endochondral ossification that takes place in the epiphyseal growth plate, a cartilaginous structure found near the ends of long bones. Evidently, many of the known monogenic growth disorders that lead to short stature involves deleterious variants of genes with important functions in the growth plate (5). In the epiphysis of an actively growing child, chondrocytes, which is the main cell type found in the growth plate, are arranged histologically into three different zones called the resting, proliferative, and hypertrophic zone. Resting zone chondrocytes function as stem cells or progenitor cells in the cartilage, capable of self-renewing and giving rise to new clones of proliferative chondrocytes right underneath the resting zone (6, 7). Cells in the proliferative zones are then arranged in columns parallel to the long axis of the bone, and these chondrocytes near the top half of the columns actively divide while gradually descend to the bottom half of the columns. Eventually, these columnar chondrocytes cease to divide and undergo hypertrophic differentiation as they approach the bottom of the growth plate. Hypertrophic chondrocytes near the bottom of the growth plate also produce matrix metalloproteinases (MMPs) and vascular endothelial growth factors (VEGFs) to resolve the mineralized cartilage matrix and initiate angiogenesis. This allows the recruitment and infiltration of osteoblasts to remodel the cartilage scaffold into bone tissue, which results in bone elongation. The terminal hypertrophic chondrocytes were initially thought to all undergo apoptosis, although lineage tracing experiments in recent years showed that many of these hypertrophic chondrocytes instead directly undergo transdifferentiation into osteoblasts, which contribute to bone formation and continue to reside in the trabecular space (8).

Genome-wide association studies (GWAS) of human stature showed that regulation of gene expression in the growth plate is instrumental in determining final adult height (9). Evidently, height-associated genomic loci are strongly enriched with genes highly expressed and spatially- or temporally-regulated in the growth plate (10), which is further supported by a recent CRISPR screening that showed enrichment of height-associated loci in genes impacting chondrocyte functions (11). In the past few years, epigenetic and transcriptome profiling of the murine growth plate (12, 13) demonstrated that the growth plate is spatially dynamic in terms of epigenetic modifications. In combination with the identification of common variants of epigenetic regulators in the height-associated GWAS and rare variants of epigenetic regulators in human growth disorders (to be discussed below), these data are likely to yield important insights into the role of epigenetics on human bone growth.

## Histone acetyltransferases, deacetylases, and chondrocyte hypertrophy

Enzymes that catalyze the acetylation of core histone proteins are known as HATs, and conversely, enzymes that catalyze the removal of histone acetylation are known as HDACs. The involvement of histone acetylation in gene regulation was theorized back in 1964, when Allfrey et al. hypothesized that the positively charged histones normally bind tightly to the negatively charge DNA through electrostatic interactions, and that acetylation adds negative charges to the histones (14), thereby weakening the histone-DNA interactions to improve accessibility of transcription factors and RNA polymerases to facilitate gene expression. Histone acetylation by HATs is therefore considered a “stimulatory” chromatin modification associated with gene expression, while removal of histone acetylation by HDACs is considered a “inhibitory” chromatin modification associated with gene suppression. Specifically in chondrocytes, expression of chondrogenic markers including *SOX9* and *COL2A1* were shown to be mediated by acetylation of histone by acetyltransferase p300 or CREB binding protein (CBP) (15). Evidently, heterozygous mutation of either *p300* or *CBP* cause a rare autosomal dominant growth disorder called Rubinstein-Taybi syndrome (16) manifested by short stature, dysmorphic facies, and intellectual disabilities, suggesting that p300/CBP-mediated histone acetylation is important for longitudinal bone growth.

**Figure 2:**
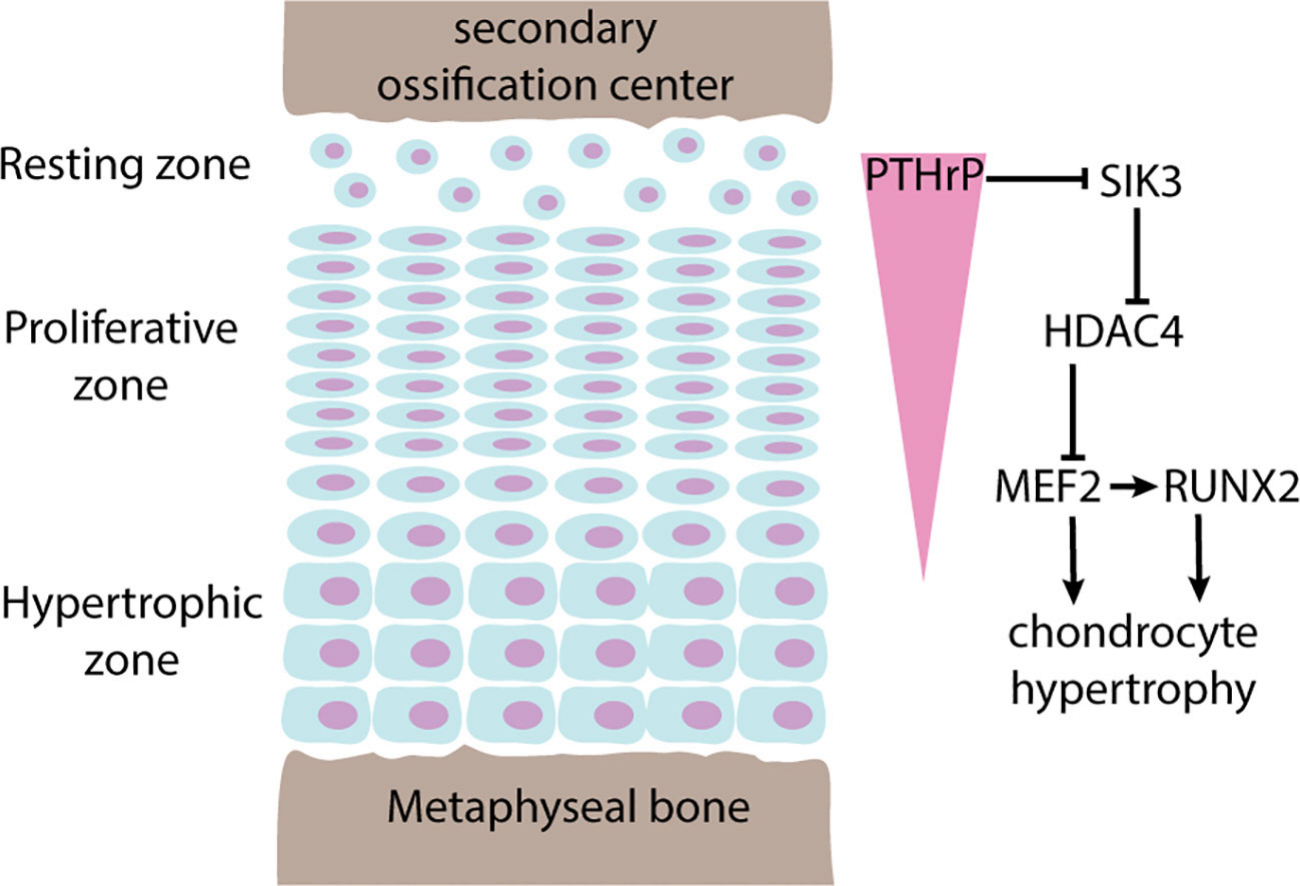
Regulation of chondrocyte hypertrophy by PTHrP-HDAC4. Pointed arrows indicate activation, blunted arrows indicate inhibition. The growth plate is spatially organized into 3 histologically distinct zones named the resting zone, proliferative zone, and hypertrophic zone. Chondrocytes in the resting zone produces parathyroid hormone related peptide (PTHrP) that diffuses across the growth plate forming a concentration gradient (pink triangle). In areas where PTHrP levels are high, chondrocyte hypertrophy is suppressed by phosphorylation and inhibition of SIK3 activity, which in turn keeps HDAC4 activated. Active HDAC4 maintains histone deacetylation and inhibition of MEF2, a transcription factor that promotes chondrocyte hypertrophy by direct actions and by activation of RUNX2.

In particular, histone acetylation and deacetylation appear to be instrumental for the spatial regulation of chondrocyte hypertrophy in the growth plate. As mentioned in the previous section, chondrocytes in different zones of the growth plate are specialized in different functions. This spatial organization is enabled and maintained by a number of paracrine gradients across the growth plate, and one prominent example is the parathyroid hormone related peptide (PTHrP) (17). PTHrP is produced in the resting zone and diffuses across the growth plate from the epiphyseal end to the metaphyseal end, forming a spatial gradient. Because PTHrP is also an inhibitor of chondrocyte hypertrophy, this PTHrP gradient also determines where chondrocyte hypertrophy begins, which becomes the boundary between proliferative zone and hypertrophic zone. Recent studies have shown that this function of PTHrP is mediated via histone deacetylase HDAC4 (18). Histone deacetylation by HDAC4 normally suppresses chondrocyte hypertrophy by blocking the expression of transcription factors *MEF2C* and *RUNX2*. Consequently, mice with *Hdac4* knockout showed premature hypertrophy and ectopic bone formation, while transgenic mice overexpressing *Hdac4* showed delayed hypertrophy and lack of bone formation (19). Interestingly, recent studies showed that HDAC4 activity is also regulated spatially by PTHrP. PTHrP suppresses salt-inducible kinase 3 (Sik3), which in turn decreases HDAC4 phosphorylation and its inhibition by 14-3-3, allowing HDAC4 activation (20). Therefore, a PTHrP concentration gradient is translated into a HDAC4 activity gradient across the growth plate to restrict activation of *MEF2C* and *RUNX2* to only the hypertrophic zone (**Figure 2**). The mechanistic connection between PTHrP and HDAC4 also help explain why *HDAC4* haploinsufficiency causes Brachydactyly mental retardation syndrome (21), which is phenotypically similar to Brachydactyly type E caused by mutations in *PTHLH*, the gene that encodes PTHrP (22). Furthermore, heterozygous loss-of-function variants of histone acetyltransferase *KAT6B* causes two different genetic disorders, Genitopatellar syndrome and SBBYSS syndrome (23), both of which are characterized by patellar hypoplasia, and a variety of growth phenotype that may include microcephaly and postnatal growth retardation, which has been proposed to be mediated by regulation of *RUNX2* expression by *KAT6B* (24).

Importantly, HDACs have been shown to deacetylate both histones and other non-histone proteins, and not all mechanistic functions of HDACs are necessarily mediated by histone deacetylation. For example, Cornelia de Lange Syndrome 5 (CdLS5) caused by variants of *HDAC8* was mediated primarily by acetylation of *SMC3*, a gene where mutation itself causes CdLS3, rather than by acetylation of histones (25).

## Histone methyltransferases, demethylases (KDM), and Kabuki syndrome

Compared to acetylation, histone methylation has a much more complicated relationship with transcriptional activation and repression. While acetylation is generally considered a histone mark associated with gene activation, histone methylation can be associated with activation or repression depending on which residue is methylated, and whether the residue is mono-, di- or tri-methylated. To add to the complexity, histones can be methylated at multiple residues, and the outcome of transcriptional regulation could be determined by the combination of multiple signals imposed by these methylations. Generally speaking, methylation of histone 3 at lysine 4 (H3K4), lysine 36 (H3K36) and lysine 79 (H3K79) are associated with transcriptional activation, while methylation of histone 3 at lysine 9 (H3K9), lysine 27 (H3K27), and histone 4 lysine 20 (H4K20) are associated with transcriptional repression (26). Histone methyltransferases and demethylases are also residue-specific in general, so for example *KMT2A* is a methyltransferase for H3K4 (27), and because H3K4 methylation is generally associated with transcriptional activation, loss-of-function mutation of *KMT2A* tend to be associated with downregulation of its target genes.

Perhaps because of such complexity of histone methylation, the molecular mechanisms by which genetic variants of methyltransferase and/or demethylase cause childhood growth disorder has so far been difficult to decipher. For example, heterozygous (primarily loss-of-function) variants of H3K4 methyltransferase *KMT2A* and *KMT2D* (also called *MLL1* and *MLL2*, respectively) cause two separate autosomal dominant growth disorders with short stature. Mutations of *KMT2A* is associated with Wiedemann-Steiner syndrome (28), while mutations of *KMT2D* is associated with Kabuki syndrome (29). The mechanism by which H3K4 methylation impacted bone growth has also been elucidated in mouse model of Kabuki syndrome. *Shox2* expression was downregulated in *Kmt2d* ^-/-^ chondrocytes, which led to upregulation of *Sox9* and in turn delay of chondrocyte hypertrophy (30). In the same study, mice with haploinsufficiency of *Kmt2d* showed decreased long bone growth, expanded growth plate and delayed endochondral ossification (30). Paradoxically however, loss-of-function variants of *KDM6A*, which is an H3K4 demethylase and therefore supposedly the functional opposite of *KMT2A* or *KMT2D*, also cause Kabuki syndrome (31, 32). A mechanistic rationale as to why genetic disruption of H3K4 methyltransferase and demethylase can both lead to Kabuki syndrome has so far been lacking.

## An epigenetic link to overgrowth syndromes

In 2017, a clinical study with more than 700 patients found that genetic variants in epigenetic regulators are a major cause of overgrowth with intellectual disability (33). This is of particular interest not only from a clinical diagnosis perspective but also from a fundamental biology perspective for a couple of reasons. Firstly, unlike short stature or growth retardation, which could sometimes be non-specific due to decrease in overall organismal fitness, overgrowth syndrome tend to be more specific about growth, and especially if it was not caused by increase in growth hormone or insulin-like growth factor I (IGF-I), could point to new biology about bone growth; and secondly, such connection between overgrowth and epigenetic regulators may imply that epigenetic modifications are involved in limitation of overall organismal body size, which has been a fascinating mystery in biology (34).

Overgrowth syndromes caused by genetic variants in epigenetic regulators can be classified into three main groups according to the epigenetic modification involved: variants that affect H3K36 histone methyltransferase, *NSD1* and *SETD2*, cause Sotos (35) and Sotos-like syndrome (now renamed as Luscan-Lumish syndrome) (36); variants in components of the polycomb repressive complex 2 (PRC2), including *EZH2, EED*, and *SUZ12*, that catalyzes H3K27 methylation cause Weaver (37) or Weaver-like syndrome (now renamed as Cohen-Gibson syndrome and Imagawa-Matsumoto syndrome) (38, 39); and variants in DNA methyltransferase *DNMT3A* cause Tatton-Brown Rahman Syndrome (40). All three syndromes are phenotypically similar, which suggest the possibility of some common yet unknown underlying molecular mechanisms that cause overgrowth (41), despite the fact that H3K36 methylation is associated with active transcription, but H3K27 methylation and DNA methylation are generally associated with gene silencing with no obvious connection between all three.

Mouse models with cartilage-specific deletion of these epigenetic regulators could provide us with some mechanistic insights into the role of these epigenetic modifications in bone growth. For example, impaired cartilage development and bone growth was observed in *Prx1* ^cre^ *Nsd1* ^f/f^ mice when *Nsd1* was knocked out in mesenchymal progenitors, where chondrocytes originated from, suggesting that *Nsd1* is important for the early stages of endochondral bone formation (42). The same study also demonstrated that *Nsd1*-mediated H3K36 methylation is needed for transcription of *Sox9* and *Hif1α*, two important transcription factors during chondrocyte differentiation (42). Similarly, cartilage-specific knockout of either *Eed* (43) or *Ezh2* (44) in mice impaired both chondrocyte proliferation and hypertrophy via a combination of signaling pathways including IGF-I, WNT, and TGF-β signaling. Although these studies highlighted the importance of H3K36 and H3K27 methylation in skeletal development and bone growth, conspicuously, they did not recapitulate the human overgrowth phenotype. To date only two mouse models relevant to these overgrowth syndromes have reported increased body growth, which are heterozygous single mis-sense loss-of-function mutations of either *EZH2* (45) or *DNMT3A* (46), suggesting that while the complete loss of these KMTs or DNMTs is detrimental, it is conceivable that a more subtle, partial reduction of activity could be growth-promoting. Several studies have tried to characterize crosstalk between *NSD1*, *PRC2*, and *DNMT3A* in an attempt to mechanistically unify these overgrowth syndromes (47, 48). Based on an hypothesis proposed by Deevy and Bracken, an intricate balance between H3K27 and H3K36 di-methylation enables *DNMT3A* localization to the intergenic region across the genome (49), which became disrupted by mutations of either *NSD1*, *EZH2*, or *DNMT3A*. Nevertheless, the connection of such balance in epigenetic modifications with body growth or bone growth has yet to be elucidated.

## Epigenetic reader: MECP2, BRD4, and SPIN4

As mentioned above, epigenetic readers are proteins that recognize and interacts with epigenetic modifications to modulate transcription. Consequently, these proteins usually contain structural domains that are known to interact with histones or methylated DNA. Methyl-CpG-binding domains (MBDs) are responsible for binding to methylated cytosines in DNA; Bromodomains are typically found in readers for histone acetylation; while Chromodomains, PHD fingers, or Tudor domains could be found in readers for histone methylation.

Inactivating mutations of DNA methylation reader protein *MECP2* is associated with Rett Syndrome, an X-linked neurodevelopmental disorder that may include microcephaly and short stature (50). Consistently, *Mecp2* knockout mouse showed significantly decreased bone length and overall skeletal size, with abnormal growth plate histology and reduced cortical, trabecular and calvarial bone volume (51). It is yet unclear exactly to what extend these growth phenotypes are driven by *MECP2* functions in chondrocyte or osteoblast without using a tissue-specific model, considering the importance of *MECP2* in neurodevelopment (52).

Although no human genetic disease has so far been ascribed to mutations of histone acetylation readers like BRD proteins, they are still of considerable interest due to their connections with disease-causing HATs and HDACs. Consistent with the role of p300 in *SOX9* transcription, *Prx1*-specific *Brd4* deletion suppresses *Sox9* expression in murine mesenchymal progenitors (53). In a separate study, bone cells treated with BRD inhibitors resulted in transcriptional silencing of *RUNX2* due to depletion of BRD4 binding (54), corroborating previous findings on *HDAC4* and *RUNX2*, and suggested further that binding of *BRD4* to acetylated histone at the *RUNX2* loci is necessary for its expression. Consequently, knocking out *Brd4* in the mesenchyme led to abnormal growth plate histology, delayed endochondral ossification and decreased long bone length (53). It would not be a surprise if causative variants of *BRD4* are eventually to be identified in patients with short stature or skeletal abnormalities.

Recently, truncating variant in *SPIN4*, a Tudor-domains containing histone methylation reader protein that can stimulate WNT signaling (55), was identified in a family with X-linked overgrowth (56). Hemizygous male patient with the *SPIN4* variant has extreme tall stature (+4.8 SD) but otherwise normal psychomotor and intellectual development, while female individuals heterozygous for the variant are also tall (+2 SD). Mice carrying truncating variants of *Spin4* developed overgrowth with increased bone length, recapitulating the growth phenotype in the family. Growth plate histology showed increased progenitor cells in the resting zone of *Spin4* mutant mice, which could be attributed to a WNT-inhibitory environment, thought to be favorable for stem cell maintenance (57), created by loss of *Spin4* expression (56). Future genetic analysis will help clarify if mutations in *SPIN4* may be more commonly found in patients with overgrowth or tall stature.

## microRNA-140 and human skeletal disorders

Non-coding RNAs (ncRNAs), including microRNAs, are sometimes viewed as distinct from epigenetic regulations. Given the growing body of literature on ncRNAs and their role in skeletal growth, a separate, comprehensive review on this topic is warranted. However, for the scope of this discussion, we will concentrate on some recent studies that are particularly relevant to human skeletal disorders.

microRNAs (miRNAs) is a type of short non-coding RNA that binds to untranslated regions of messenger RNA of target genes to inhibit expression, and its importance in bone growth have long been demonstrated in mice. For example, cartilage-specific knockout of *Dicer*, the enzyme essential for miRNA biogenesis, showed lethal skeletal growth failure with decreased chondrocyte proliferation and accelerated hypertrophy (58). Amongst the many miRNAs abundantly expressed in chondrocytes, miR-140 was the first being shown to have important functions in the growth plate. Knockout of *miR140* resulted in short stature and craniofacial abnormalities in mice (59, 60). In these mice, an expansion of resting zone in the growth plate was observed, suggesting that miR-140 is important for the transition of resting zone progenitor cells into proliferative chondrocytes (60, 61). Subsequently, many more studies have demonstrated the role of miRNAs in chondrocyte functions *in vitro* (62), although for many years the clinical implications in human growth disorders have been lacking. Finally in 2019, gain-of-function variants of *miR140* was identified in three patients from two independent families with a novel skeletal dysplasia characterized by disproportionate short stature with short limbs, small hands and feet (the syndrome was subsequently named Spondyloepiphyseal dysplasia Nakamura type) (63), providing the definitive genetic proof that *miR140*, if not miRNA in general, is important for human growth plate physiology.

## Future perspectives: potential and challenges for epidrugs

The exceptional progress being made in the past decade on clinical genetics and disease mechanisms have led to major advancements in our understanding in epigenetic regulations in childhood bone development. However, we are still at the beginning of a new technological epoch in translating these knowledges into therapeutic interventions. Compared to genetic mutations, epigenetic alternations have greater plasticity and are more likely to be reversible, such as in the case of induced pluripotent stem cells (iPSCs) (64).

Epigenetic drugs, or epidrugs, are small molecules or chemical compounds that mainly work as an inhibitor or activator of enzymes like histone acetyltransferases or demethylases to modulate the epigenetic modifications, aiming to restore normal epigenomic landscape to correct cellular functions and organismal phenotypes. Epidrug design and experimentation is a growing field for drug discovery in cancer biology, with a handful of therapeutics already approved by FDA (**Table 2**) (65). For example, DNMT inhibitor 5-azacitidine (or Vidaza) is approved for myelodysplastic syndrome (66); HDAC inhibitors Vorinostat is approved for T-cell lymphoma (67); and more recently, EZH2 inhibitor Tazemetostat (or Tazverik) is approved for follicular lymphoma and epithelioid sarcoma (68). Could any of these epidrugs for cancer therapy be repurposed for childhood growth or skeletal disorders since they work on the same epigenetic regulators? The potential is certainly appealing although perhaps major concerns for unwanted adverse and off-target effects cannot be overlooked. To complicate things further, there appears to be an optimal epigenetic state where any deviation or disruption of the equilibrium might be unfavorable, such that for example mutations of H3K4 methyltransferase and demethylase both cause Kabuki syndrome (29, 31), and *miR140* gain-of-function (Spondyloepiphyseal dysplasia) and loss-of-function (in mouse models) both resulted in short stature (60, 63). Therapeutics might have to strike the right balance between activation and inhibition to achieve optimal outcomes. Using Weaver syndrome as another example, a subtle decrease of EZH2 activity appeared to promote long bone growth (45) but further reduction of EZH2 activity may lead to growth retardation (44). In theory, one could try to pharmacologically modulate EZH2 activity to stimulate bone growth, but what is the optimal EZH2 activity for growth stimulation? The therapeutic window here could be too narrow for drug development to be palatable, even if we were to fully understand how EZH2 activity is mechanistically linked to bone growth. At the current stage, a more thorough understanding of epigenetic regulations of bone growth is still a necessity before seeing epidrug blossom into the next frontier of skeletal/growth therapeutics.

**Table 2:** List of FDA-approved epidrugs.

Generic name	Brand name (company)	Indication (s)	Mechanism of action	Year approved
Vorinostat	Zolinza (Merck & Co.)	Cutaneous T cell lymphoma	HDAC inhibitor	2006
Decitabine	Dacogen (MGI Pharma)	Myelodysplastic Diseases	DNMT inhibitor	2006
Azacitidine	Vidaza (Pharmion)	Myelodysplastic Diseases	DNMT inhibitor	2009
	Onureg (BMS)	Acute Myeloid Leukemia		2020
Romidepsin	Istodax (Gloucester)	Cutaneous T cell lymphoma	HDAC inhibitor	2009
	Istodax (BMS)	Peripheral T cell lymphomas (withdrawn 2021)		2011
Valproic Acid	Depacon, Depakene, etc (Abbott, others)	Epilepsy, Bipolar mania, Migraine prophylaxis	HDAC inhibitor	2010
Belinostat	Beleodaq (Spectrum)	Peripheral T cell lymphomas	HDAC inhibitor	2014
Panobinostat	Farydak (Secura Bio)	Multiple Myeloma (discontinued 2021)	HDAC inhibitor	2015
Tazemetostat	Tazverik (Epizyme)	Epithelioid Sarcoma; Follicular Lymphoma	EZH2 inhibitor	2020

## Author contributions

JL: Conceptualization, Data curation, Formal analysis, Funding acquisition, Resources, Validation, Visualization, Writing – original draft, Writing – review & editing.

## References

[1] Hojo H Ohba S. Gene regulatory landscape in osteoblast differentiation. Bone (2020) 137:115458. doi: 10.1016/j.bone.2020.115458 32474244

[2] Wang J Shi A Lyu J. A comprehensive atlas of epigenetic regulators reveals tissue-specific epigenetic regulation patterns. Epigenetics (2023) 18:2139067. doi: 10.1080/15592294.2022.2139067 36305095 PMC9980636

[3] Hyun K Jeon J Park K Kim J. Writing, erasing and reading histone lysine methylations. Exp Mol Med (2017) 49:e324. doi: 10.1038/emm.2017.11 28450737 PMC6130214

[4] Geister KA Camper SA. Advances in skeletal dysplasia genetics. Annu Rev Genomics Hum Genet (2015) 16:199–227. doi: 10.1146/annurev-genom-090314-045904 25939055 PMC5507692

[5] Jee YH Andrade AC Baron J Nilsson O. Genetics of short stature. Endocrinol Metab Clin North Am (2017) 46:259–81. doi: 10.1016/j.ecl.2017.01.001 PMC5424617 28476223

[6] Abad V Meyers JL Weise M Gafni RI Barnes KM Nilsson O. The role of the resting zone in growth plate chondrogenesis. Endocrinology (2002) 143:1851–7. doi: 10.1210/endo.143.5.8776 11956168

[7] Lui JC. Home for a rest: stem cell niche of the postnatal growth plate. J Endocrinol (2020) 246:R1–r11. doi: 10.1530/JOE-20-0045 32240983 PMC7237297

[8] Yang L Tsang KY Tang HC Chan D Cheah KS. Hypertrophic chondrocytes can become osteoblasts and osteocytes in endochondral bone formation. Proc Natl Acad Sci USA (2014) 111:12097–102. doi: 10.1073/pnas.1302703111 PMC4143064 25092332

[9] Wood AR Esko T Yang J Vedantam S Pers TH Gustafsson S. Defining the role of common variation in the genomic and biological architecture of adult human height. Nat Genet (2014) 46:1173–86. doi: 10.1038/ng.3097 PMC4250049 25282103

[10] Lui JC Nilsson O Chan Y Palmer CD Andrade AC Hirschhorn JN. Synthesizing genome-wide association studies and expression microarray reveals novel genes that act in the human growth plate to modulate height. Hum Mol Genet (2012) 21:5193–201. doi: 10.1093/hmg/dds347 PMC3490510 22914739

[11] Baronas JM Bartell E Eliasen A Doench JG Yengo L Vedantam S. Genome-wide CRISPR screening of chondrocyte maturation newly implicates genes in skeletal growth and height-associated GWAS loci. Cell Genom (2023) 3:100299. doi: 10.1016/j.xgen.2023.100299 37228756 PMC10203046

[12] Guo M Liu Z Willen J Shaw CP Richard D Jagoda E. Epigenetic profiling of growth plate chondrocytes sheds insight into regulatory genetic variation influencing height. Elife (2017) 6. doi: 10.7554/eLife.29329 PMC5716665 29205154

[13] Wuelling M Neu C Thiesen AM Kitanovski S Cao Y Lange A. Epigenetic mechanisms mediating cell state transitions in chondrocytes. J Bone Miner Res (2021) 36:968–85. doi: 10.1002/jbmr.4263 33534175

[14] Allfrey VG Faulkner R Mirsky AE. Acetylation and methylation of histones and their possible role in the regulation in the RNA synthesis. Proc Natl Acad Sci USA (1964) 51:786–94. doi: 10.1073/pnas.51.5.786 PMC300163 14172992

[15] Furumatsu T Tsuda M Yoshida K Taniguchi N Ito T Hashimoto M. Sox9 and p300 cooperatively regulate chromatin-mediated transcription *. J Biol Chem (2005) 280:35203–8. doi: 10.1074/jbc.M502409200 16109717

[16] Petrij F Giles RH Dauwerse HG Saris JJ Hennekam RC Masuno M. Rubinstein-Taybi syndrome caused by mutations in the transcriptional co-activator CBP. Nature (1995) 376:348–51. doi: 10.1038/376348a0 7630403

[17] Vortkamp A Lee K Lanske B Segre GV Kronenberg HM Tabin CJ. Regulation of rate of cartilage differentiation by Indian hedgehog and PTH-related protein. Science (1996) 273:613–22. doi: 10.1126/science.273.5275.613 8662546

[18] Nishimori S Lai F Shiraishi M Kobayashi T Kozhemyakina E Yao TP. PTHrP targets HDAC4 and HDAC5 to repress chondrocyte hypertrophy. JCI Insight (2019) 4. doi: 10.1172/jci.insight.97903 PMC6483522 30843886

[19] Vega RB Matsuda K Oh J Barbosa AC Yang X Meadows E. Histone deacetylase 4 controls chondrocyte hypertrophy during skeletogenesis. Cell (2004) 119:555–66. doi: 10.1016/j.cell.2004.10.024 15537544

[20] Nishimori S Wein MN Kronenberg HM. PTHrP targets salt-inducible kinases, HDAC4 and HDAC5, to repress chondrocyte hypertrophy in the growth plate. Bone (2021) 142:115709. doi: 10.1016/j.bone.2020.115709 33148508 PMC7744326

[21] Williams SR Aldred MA Der Kaloustian VM Halal F Gowans G Mcleod DR. Haploinsufficiency of HDAC4 causes brachydactyly mental retardation syndrome, with brachydactyly type E, developmental delays, and behavioral problems. Am J Hum Genet (2010) 87:219–28. doi: 10.1016/j.ajhg.2010.07.011 PMC2917703 20691407

[22] Klopocki E Hennig BP Dathe K Koll R De Ravel T Baten E. Deletion and point mutations of PTHLH cause brachydactyly type E. Am J Hum Genet (2010) 86:434–9. doi: 10.1016/j.ajhg.2010.01.023 PMC2833367 20170896

[23] Campeau PM Lu JT Dawson BC Fokkema IF Robertson SP Gibbs RA. The KAT6B-related disorders genitopatellar syndrome and Ohdo/SBBYS syndrome have distinct clinical features reflecting distinct molecular mechanisms. Hum Mutat (2012) 33:1520–5. doi: 10.1002/humu.22141 PMC3696352 22715153

[24] Ullah M Pelletier N Xiao L Zhao SP Wang K Degerny C. Molecular architecture of quartet MOZ/MORF histone acetyltransferase complexes. Mol Cell Biol (2008) 28:6828–43. doi: 10.1128/MCB.01297-08 PMC2573306 18794358

[25] Deardorff MA Bando M Nakato R Watrin E Itoh T Minamino M. HDAC8 mutations in Cornelia de Lange syndrome affect the cohesin acetylation cycle. Nature (2012) 489:313–7. doi: 10.1038/nature11316 PMC3443318 22885700

[26] Whetstine JR. “Chapter 287 - histone methylation: chemically inert but chromatin dynamic”. In: Bradshaw RA Dennis EA, editors. Handbook of Cell Signaling, 2nd ed. San Diego: Academic Press (2010). p. 2389–97.

[27] Krivtsov AV Armstrong SA. MLL translocations, histone modifications and leukaemia stem-cell development. Nat Rev Cancer (2007) 7:823–33. doi: 10.1038/nrc2253 17957188

[28] Jones WD Dafou D Mcentagart M Woollard WJ Elmslie FV Holder-Espinasse M. *De novo* mutations in MLL cause Wiedemann-Steiner syndrome. Am J Hum Genet (2012) 91:358–64. doi: 10.1016/j.ajhg.2012.06.008 PMC3415539 22795537

[29] Ng SB Bigham AW Buckingham KJ Hannibal MC Mcmillin MJ Gildersleeve HI. Exome sequencing identifies MLL2 mutations as a cause of Kabuki syndrome. Nat Genet (2010) 42:790–3. doi: 10.1038/ng.646 PMC2930028 20711175

[30] Fahrner JA Lin WY Riddle RC Boukas L Deleon VB Chopra S. Precocious chondrocyte differentiation disrupts skeletal growth in Kabuki syndrome mice. JCI Insight (2019) 4. doi: 10.1172/jci.insight.129380 PMC6824315 31557133

[31] Van Laarhoven PM Neitzel LR Quintana AM Geiger EA Zackai EH Clouthier DE. Kabuki syndrome genes KMT2D and KDM6A: functional analyses demonstrate critical roles in craniofacial, heart and brain development. Hum Mol Genet (2015) 24:4443–53. doi: 10.1093/hmg/ddv180 PMC4492403 25972376

[32] Bögershausen N Gatinois V Riehmer V Kayserili H Becker J Thoenes M. Mutation update for kabuki syndrome genes KMT2D and KDM6A and further delineation of X-linked kabuki syndrome subtype 2. Hum Mutat (2016) 37:847–64. doi: 10.1002/humu.23026 27302555

[33] Tatton-Brown K Loveday C Yost S Clarke M Ramsay E Zachariou A. Mutations in epigenetic regulation genes are a major cause of overgrowth with intellectual disability. Am J Hum Genet (2017) 100:725–36. doi: 10.1016/j.ajhg.2017.03.010 PMC5420355 28475857

[34] Lui JC Baron J. Mechanisms limiting body growth in mammals. Endocr Rev (2011) 32:422–40. doi: 10.1210/er.2011-0001 PMC3365796 21441345

[35] Kurotaki N Imaizumi K Harada N Masuno M Kondoh T Nagai T. Haploinsufficiency of NSD1 causes Sotos syndrome. Nat Genet (2002) 30:365–6. doi: 10.1038/ng863 11896389

[36] Luscan A Laurendeau I Malan V Francannet C Odent S Giuliano F. Mutations in SETD2 cause a novel overgrowth condition. J Med Genet (2014) 51:512–7. doi: 10.1136/jmedgenet-2014-102402 24852293

[37] Gibson WT Hood RL Zhan SH Bulman DE Fejes AP Moore R. Mutations in EZH2 cause Weaver syndrome. Am J Hum Genet (2012) 90:110–8. doi: 10.1016/j.ajhg.2011.11.018 PMC3257956 22177091

[38] Prokopuk L Stringer JM White CR Vossen R White SJ Cohen ASA. Loss of maternal EED results in postnatal overgrowth. Clin Epigenet (2018) 10:95. doi: 10.1186/s13148-018-0526-8 PMC6045828 30005706

[39] Imagawa E Seyama R Aoi H Uchiyama Y Marcarini BG Furquim I. Imagawa-Matsumoto syndrome: SUZ12-related overgrowth disorder. Clin Genet (2023) 103:383–91. doi: 10.1111/cge.14296 36645289

[40] Tatton-Brown K Seal S Ruark E Harmer J Ramsay E Del Vecchio Duarte S. Mutations in the DNA methyltransferase gene DNMT3A cause an overgrowth syndrome with intellectual disability. Nat Genet (2014) 46:385–8. doi: 10.1038/ng.2917 PMC3981653 24614070

[41] Lui JC Baron J. Epigenetic causes of overgrowth syndromes. J Clin Endocrinol Metab (2024) 109:312–20. doi: 10.1210/clinem/dgad420 PMC11032252 37450557

[42] Shao R Zhang Z Xu Z Ouyang H Wang L Ouyang H. H3K36 methyltransferase NSD1 regulates chondrocyte differentiation for skeletal development and fracture repair. Bone Res (2021) 9:30. doi: 10.1038/s41413-021-00148-y 34099628 PMC8185073

[43] Mirzamohammadi F Papaioannou G Inloes JB Rankin EB Xie H Schipani E. Polycomb repressive complex 2 regulates skeletal growth by suppressing Wnt and TGF-β signalling. Nat Commun (2016) 7:12047. doi: 10.1038/ncomms12047 27329220 PMC4917962

[44] Lui JC Garrison P Nguyen Q Ad M Keembiyehetty C Chen W. EZH1 and EZH2 promote skeletal growth by repressing inhibitors of chondrocyte proliferation and hypertrophy. Nat Commun (2016) 7:13685. doi: 10.1038/ncomms13685 27897169 PMC5477487

[45] Lui JC Barnes KM Dong L Yue S Graber E Rapaport R. Ezh2 mutations found in the weaver overgrowth syndrome cause a partial loss of H3K27 histone methyltransferase activity. J Clin Endocrinol Metab (2018) 103:1470–8. doi: 10.1210/jc.2017-01948 PMC6276576 29244146

[46] Smith AM Lavalle TA Shinawi M Ramakrishnan SM Abel HJ Hill CA. Functional and epigenetic phenotypes of humans and mice with DNMT3A Overgrowth Syndrome. Nat Commun (2021) 12:4549. doi: 10.1038/s41467-021-24800-7 34315901 PMC8316576

[47] Streubel G Watson A Jammula SG Scelfo A Fitzpatrick DJ Oliviero G. The H3K36me2 methyltransferase nsd1 demarcates PRC2-mediated H3K27me2 and H3K27me3 domains in embryonic stem cells. Mol Cell (2018) 70:371–379.e375. doi: 10.1016/j.molcel.2018.02.027 29606589

[48] Weinberg DN Papillon-Cavanagh S Chen H Yue Y Chen X Rajagopalan KN. The histone mark H3K36me2 recruits DNMT3A and shapes the intergenic DNA methylation landscape. Nature (2019) 573:281–6. doi: 10.1038/s41586-019-1534-3 PMC6742567 31485078

[49] Deevy O Bracken AP. PRC2 functions in development and congenital disorders. Development (2019) 146. doi: 10.1242/dev.181354 PMC6803372 31575610

[50] Tarquinio DC Motil KJ Hou W Lee HS Glaze DG Skinner SA. Growth failure and outcome in Rett syndrome: specific growth references. Neurology (2012) 79:1653–61. doi: 10.1212/WNL.0b013e31826e9a70 PMC3468773 23035069

[51] O'connor RD Zayzafoon M Farach-Carson MC Schanen NC. Mecp2 deficiency decreases bone formation and reduces bone volume in a rodent model of Rett syndrome. Bone (2009) 45:346–56. doi: 10.1016/j.bone.2009.04.251 PMC2739100 19414073

[52] Gonzales ML Lasalle JM. The role of MeCP2 in brain development and neurodevelopmental disorders. Curr Psychiatry Rep (2010) 12:127–34. doi: 10.1007/s11920-010-0097-7 PMC2847695 20425298

[53] Paradise CR Galvan ML Pichurin O Jerez S Kubrova E Dehghani SS. Brd4 is required for chondrocyte differentiation and endochondral ossification. Bone (2022) 154:116234. doi: 10.1016/j.bone.2021.116234 34700039 PMC9014208

[54] Lamoureux F Baud'huin M Rodriguez Calleja L Jacques C Berreur M Rédini F. Selective inhibition of BET bromodomain epigenetic signalling interferes with the bone-associated tumour vicious cycle. Nat Commun (2014) 5:3511. doi: 10.1038/ncomms4511 24646477

[55] Su X Zhu G Ding X Lee SY Dou Y Zhu B. Molecular basis underlying histone H3 lysine-arginine methylation pattern readout by Spin/Ssty repeats of Spindlin1. Genes Dev (2014) 28:622–36. doi: 10.1101/gad.233239.113 PMC3967050 24589551

[56] Lui JC Wagner J Zhou E Dong L Barnes KM Jee YH. Loss-of-function variant in SPIN4 causes an X-linked overgrowth syndrome. JCI Insight (2023) 8. doi: 10.1172/jci.insight.167074 PMC10243798 36927955

[57] Hallett SA Matsushita Y Ono W Sakagami N Mizuhashi K Tokavanich N. Chondrocytes in the resting zone of the growth plate are maintained in a Wnt-inhibitory environment. Elife (2021) 10. doi: 10.7554/eLife.64513 PMC8313235 34309509

[58] Kobayashi T Lu J Cobb BS Rodda SJ Mcmahon AP Schipani E. Dicer-dependent pathways regulate chondrocyte proliferation and differentiation. Proc Natl Acad Sci USA (2008) 105:1949–54. doi: 10.1073/pnas.0707900105 PMC2538863 18238902

[59] Miyaki S Sato T Inoue A Otsuki S Ito Y Yokoyama S. MicroRNA-140 plays dual roles in both cartilage development and homeostasis. Genes Dev (2010) 24:1173–85. doi: 10.1101/gad.1915510 PMC2878654 20466812

[60] Nakamura Y Inloes JB Katagiri T Kobayashi T. Chondrocyte-specific microRNA-140 regulates endochondral bone development and targets Dnpep to modulate bone morphogenetic protein signaling. Mol Cell Biol (2011) 31:3019–28. doi: 10.1128/MCB.05178-11 PMC3133397 21576357

[61] Papaioannou G Inloes JB Nakamura Y Paltrinieri E Kobayashi T. let-7 and miR-140 microRNAs coordinately regulate skeletal development. Proc Natl Acad Sci USA (2013) 110:E3291–3300. doi: 10.1073/pnas.1302797110 PMC3761644 23940373

[62] Shvedova M Kobayashi T. MicroRNAs in cartilage development and dysplasia. Bone (2020) 140:115564. doi: 10.1016/j.bone.2020.115564 32745689 PMC7502492

[63] Grigelioniene G Suzuki HI Taylan F Mirzamohammadi F Borochowitz ZU Ayturk UM. Gain-of-function mutation of microRNA-140 in human skeletal dysplasia. Nat Med (2019) 25:583–90. doi: 10.1038/s41591-019-0353-2 PMC6622181 30804514

[64] Papp B Plath K. Epigenetics of reprogramming to induced pluripotency. Cell (2013) 152:1324–43. doi: 10.1016/j.cell.2013.02.043 PMC3602907 23498940

[65] Miranda Furtado CL Dos Santos Luciano MC Silva Santos RD Furtado GP Moraes MO Pessoa C. Epidrugs: targeting epigenetic marks in cancer treatment. Epigenetics (2019) 14:1164–76. doi: 10.1080/15592294.2019.1640546 PMC6791710 31282279

[66] Cogle CR Scott BL Boyd T Garcia-Manero G. Oral azacitidine (CC-486) for the treatment of myelodysplastic syndromes and acute myeloid leukemia. Oncologist (2015) 20:1404–12. doi: 10.1634/theoncologist.2015-0165 PMC4679081 26463870

[67] Mann BS Johnson JR Cohen MH Justice R Pazdur R. FDA approval summary: vorinostat for treatment of advanced primary cutaneous T-cell lymphoma. Oncologist (2007) 12:1247–52. doi: 10.1634/theoncologist.12-10-1247 17962618

[68] Von Keudell G Salles G. The role of tazemetostat in relapsed/refractory follicular lymphoma. Ther Adv Hematol (2021) 12:20406207211015882. doi: 10.1177/20406207211015882 34104370 PMC8165870

